# Novel Enzyme-Free Multifunctional Bentonite/Polypyrrole/Silver Nanocomposite Sensor for Hydrogen Peroxide Detection over a Wide pH Range

**DOI:** 10.3390/s19204442

**Published:** 2019-10-14

**Authors:** Khouloud Jlassi, Mostafa H. Sliem, Kamel Eid, Igor Krupa, Mohamed M. Chehimi, Aboubakr M. Abdullah

**Affiliations:** 1 Center for Advanced Materials, Qatar University, Doha 2713, Qatar; mostafa@qu.edu.qa (M.H.S.); kamelame@outlook.com (K.E.); igor.krupa@qu.edu.qa (I.K.); 2 University Paris Est, CNRS, UMR7182, ICMPE, UPEC, F-94320 Thais, France; chehimi@icmpe.cnrs.fr

**Keywords:** bentonite clay, pyrrole, silver nanoparticles, H_2_O_2_ detection

## Abstract

Precise designs of low-cost and efficient catalysts for the detection of hydrogen peroxide (H_2_O_2_) over wide ranges of pH are important in various environmental applications. Herein, a versatile and ecofriendly approach is presented for the rational design of ternary bentonite-silylpropyl-polypyrrole/silver nanoarchitectures (denoted as BP-PS-PPy/Ag) via the in-situ photo polymerization of pyrrole with salinized bentonite (BP-PS) in the presence of silver nitrate. The Pyrrolyl-functionalized silane (PS) is used as a coupling agent for tailoring the formation of highly exfoliated BP-PS-PPy sheet-like nanostructures ornamented with monodispersed Ag nanoparticles (NPs). Taking advantage of the combination between the unique physicochemical properties of BP-PS-PPy and the outstanding catalytic merits of Ag nanoparticles (NPs), the as-synthesized BP-PS-PPy/Ag shows a superior electrocatalytic reduction and high-detection activity towards H_2_O_2_ under different pH conditions (from 3 to 10). Intriguingly, the UV-light irradiation significantly enhances the electroreduction activity of H_2_O_2_ substantially, compared with the dark conditions, due to the high photoelectric response properties of Ag NPs. Moreover, BP-PS-PPy/Ag achived a quick current response with a detection limit at 1 μM within only 1 s. Our present approach is green, facile, scalable and renewable.

## 1. Introduction

Hydrogen peroxide (H_2_O_2_) is important in myriad industrial, environmental remediation, biological, and pharmaceutical applications [1,2,3,4,5,6,7,8]. Moreover, H_2_O_2_ is ubiquitously generated as a by-product during cholesterol [9], glucose [10], glutamate [11], and lactate [12] oxidation processes. Numerous efforts are spent to develop efficient analytical methods for the detection of H_2_O_2_, namely, high-pressure liquid chromatography, colorimetric, positron emission tomography, electrochemical, bioluminescence and chemiluminescence [13,14,15,16,17]. Unlike these approaches, the electrochemical methods [18] have various advantages, including the low cost, safety, simplicity, accuracy, fast response, and high sensitivity, which are essential for the practical applications [19]. Noble metals-based catalysts are well-imminent with their outstanding electrocatalytic activity and sensitivity towards enzymtic-free H_2_O_2_ detection [20]. Among these noble metals, silver nanoparticles (Ag NPs) have unique optical, catalytic, and anti-bacterial properties [21,22,23,24]. Furthermore, the great abundance in the nature of Ag makes it more feasible for large-scale applications. Moreover, Ag NPs can provide oxygenated species facilitates the O-H splitting at low potential along with high tolerance for the reaction intermediates. This is in addition to the unique ability of Ag to disproportionate H_2_O_2_ to form O_2_ and H_2_O under low potential [25]. Generally, the electroreduction activities of H_2_O_2_ on Ag NPs were found to be size-, morphology-, and composition-dependent [26]. Incorporation of Ag NPs into conducting polymers, especially polypyrrole (PPy), is another robust roadmap for boosting the H_2_O_2_ reduction activity, owing to their synergetic physicochemical properties [27]. Furthermore, the great electrical conductivity, compatibility, redox properties, low specific density, and long-term stability of PPy can enhance the conductivity and electron mobility during the H_2_O_2_ reduction [28]. Meanwhile, the drastic electronic interaction of PPy/Ag NPs can enhance the mass transfer and accelerate the reduction kinetics of H_2_O_2_. Thus, various methods were developed for the controlled synthesis of PPy/Ag nanocomposites with different morphologies [29,30,31,32,33]. However, the performances of PPy/Ag toward H_2_O_2_ reduction were not emphasized enough compared to other applications. For instance, Ag NP-decorated PPy prepared by the electrochemical method displayed a substantial electroreduction activity towards H_2_O_2_ detection with a minimum detection limit.

Combining natural purified bentonite clay (BP) with PPy/Ag as a ternary nanocomposite (BP-PPy/Ag) can exhibit remarkable physicochemical and responsive properties towards various catalytic reactions [34]. This is originated from the intrinsic high-surface area to the volume ratio, and rich electron density of BP. In addition, the cation exchange capacity of BP can enhance its electronic interaction with PPy/Ag. It is noteworthy that, the facile, low-cost and UV-induced preparation of BP-PPy/Ag remains a grand challenge and is rarely reported [35]. The latter was tested for antibacterial [36], electromagnetic shielding [37], and catalytic applications [38]. Meanwhile, the H_2_O_2_ reduction and/or detection performance on BP-PPy/Ag is not yet reported to the best of our knowledge.

In pursuit of this aim, we present herein, a facile, scalable, versatile approach for rational one-pot fabrication of bentonite-silylpropyl-polypyrrole/silver (BP-PS-PPy/Ag) with high-yield via the in-situ photo polymerization of pyrrole with the assistance of silver nitrate in the presence of salinized bentonite (BP-PS). This is beneficial for the excellent dispersion ability of silanized bentonite in an aqueous solution to form a stable colloidal network and the photosynthetic activity of silver. Unlike previous reports on BP-Ag and/or PPy/Ag nanocomposites, our approach is ecofriendly, simple, and allows the fabrication of high-exfoliated BP-PS-PPy nanosheets ornamented with monodispersed Ag NPs without additional steps for functionalization and/or activation. Furthermore, our newly developed BP-PS-PPy/Ag nanocomposite combines between the unique physicochemical properties of BP-PS-PPy (i.e., large surface area, conductivity, redox, and long-term stability) and the catalytic merits of Ag NPs (the strong dissociative ability of O-H bond in H_2_O_2_ and the high-tolerance for the reaction intermediates). These structure and composition features led to a substantial enhancement in the electrocchemcial and photoelectrochemcial H_2_O_2_ reduction activity of the as-synthesized BP-PS-PPy/Ag relative to metal-free BP-PS-PPy over a wide range of pH conditions (3, 5, and 7).

## 2. Experimental

### 2.1. Chemicals and Materials

Pyrrole ((Py) 98%) and silver nitrate ((AgNO_3_) 99.9%) were obtained from Sigma-Aldrich Chemie GmbH (Munich, Germany). N-(3-Trimethoxysilylpropyl) pyrrole ((PS) 95.0%) and H_2_O_2_ (30%) were provided from Alfa Aesar (Smithtown, NY, USA). The raw bentonite was extracted from the Gafsa-Metloui basin (Tunisia); the longitude, Lambert coordinates is about 6 graduates and 69 min (East) and the latitude of 38 degrees and 27 min, (North). The purified bentonite (noted BP) was obtained by a standard procedure reported previously [39].

### 2.2. Preparation of Bentonite-Silylpropyl-Polypyrrole/Silver (BP-PS-PPy/Ag) Nanoarchitecture

BP-PS was initially prepared via mixing 2 g of BP and 114.8 mg of PS in an aqueous solution containing 100 mL H_2_O and 10 mL ethanol/acetic acid (95/5) under stirring for 48 h at room temperature. The BP-PS was purified by centrifugation at 10,000 rpm and washing cycles for 4 times and then dried at 40 °C for 48 h. Following that, a 1 g of as-obtained BP-PS was dispersed in 50 mL ethanol and then 10 mL of H_2_O containing 1.68 g of AgNO_3_ was quickly added under stirring at room temperature followed by a dropwise of Pyrrole (20 mL, 0.5 mol/L) under the UV-illumination at 365 nm for 2 h. Finally, BP-PS-PPy/Ag nanoarchitectures were obtained via centrifugation at 7000 rpm for 10 min and washing cycles with ethanol for 4 times and then dried at 40 °C for 24 h and kept for further characterizations.

### 2.3. Materials Characterization

The morphologies of the as-formed BP-PS-PPY/Ag and BP-PS nanostructure were investigated by a transmission electron microscope (TEM TecnaiG220, FEI, Hillsboro, OR, USA). The X-ray diffraction pattern (XRD) was measured on an X’Pert-Pro MPD diffractometer (PANalytical Co., Almelo, The Netherlands) using Cu Kα X-ray source (λ = 1.540598 Å). The X-ray photoelectron spectroscopy (XPS) was analyzed on a Kratos Axis Ultra XPS spectrometer (Kratos, Manchester, UK) equipped with a monochromatic Al Kα radiation source (1486.6 eV) in a UHV environment (ca. 5 × 10^−9^ Torr). The Fourier transformed infrared spectra (FTIR) was measured on (Thermo Scientific, Madison, WT, USA). 

### 2.4. Electrocatalytic Reduction of Hydrogen Peroxide (H_2_O_2_)

The cyclic voltammograms (CVs) and chronoamperometric measurements were measured on a Gamry electrochemical analyzer (reference 3000, Gamry Co., Warminster, PA, USA), using a three-electrode cell, including a platinum wire, Ag/AgCl, glassy carbon (GC, 5 mm) as a counter, reference and working electrodes, respectively. The GC electrodes were covered with 10 µL of each catalyst followed by the addition of 5 μL Nafion (0.05%) and left to be fully dried before the measurements. For the photocatalytic reaction, a three-electrode photo-glass cell was used, and the light source was Biogro ozone-free xenon lamp (100 mW/cm^2^, HK, China). The CVs measurements of each catalyst were tested in aqueous solutions of saline phosphate buffer (pH 7.4) at 50 mV s^−1^ without and with an aqoues solution of H_2_O_2_ (20 μM). The tested solutions were deareated by purging high purity nitrogen gas for 30 min prior to the experiment.

## 3. Results and Discussion

**Figure 1:**
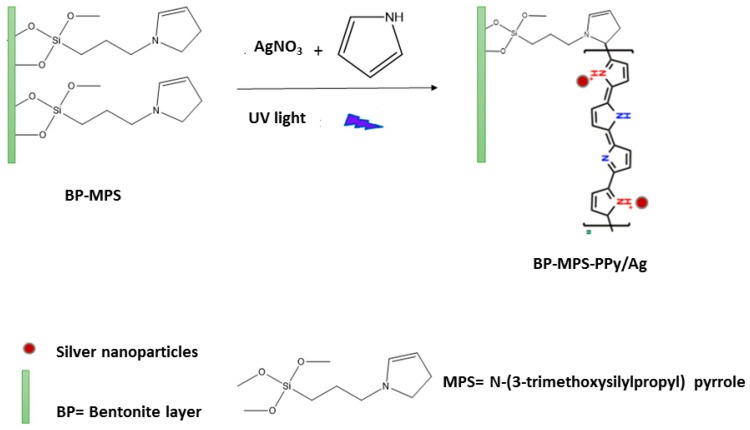
Surface treatment of purified Bentonite (BP) by N-(3-trimethoxysilylpropyl) pyrrole coupling agent followed by photopolymerization of polypyrrole (PPy)/silver (Ag).

Figure 1 illustrates the sequential steps for preparing the BP-PS-PPY/Ag ternary hybrids through two steps: First BP is grafted with amine groups using 3-aminopropyltriethoxysilane, then, pyrrole and AgNO_3_ are added to the suspension prior to exposure to the UV light under mixing.

**Figure 2:**
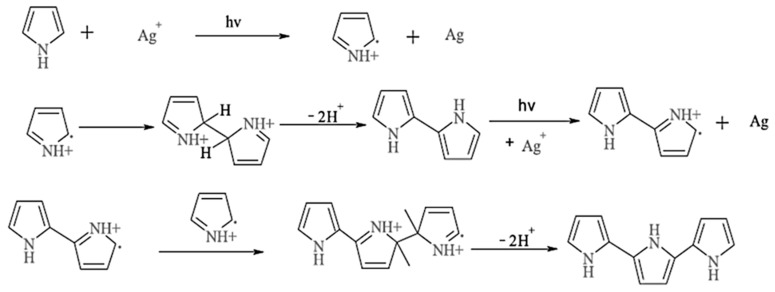
Photoplymerization mechanism of pyrrole.

The N-rich PPy can easily accelerate the photoreduction of AgNO_3_ to form Ag NPs and allow their anchoring on N-atoms of pyrrole during the photopolymerization step. This led to the in-situ formation of monodispersed Ag NPs on the surface of the resulting BP-PS-PPY hybrid nanocomposite. Figure 2 shows the detailed photopolymerization mechanism of pyrrole. First, the excited state of Ag^+^ strips an electron from pyrrole resulting in the formation of pyrrole radical cations and the reduction of Ag^+^ to metal. Two of these radical cations then couple to a dimer (dimerization) with deprotonation, leading to a bipyrrole. After the deprotonation, the bipyrrole is reoxidized and couples with another radical cation. These radical cations can react with pyrrole radical cations to form the PPy chain (chain growth) [40].

**Figure 3:**
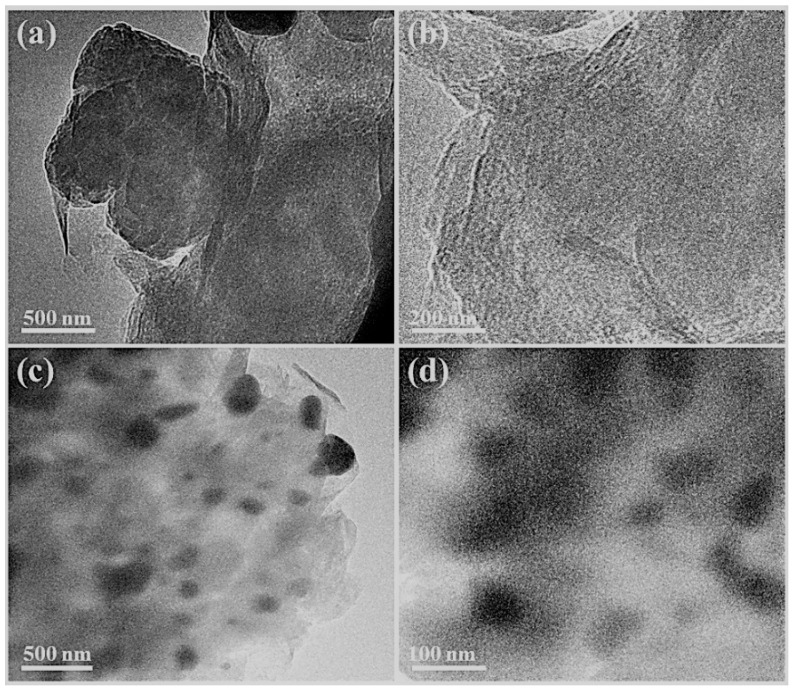
TEM images of BP-PS-PPy (**a**,**b**) and BP-PS-PPy /Ag (**c**,**d**).

**Figure 4:**
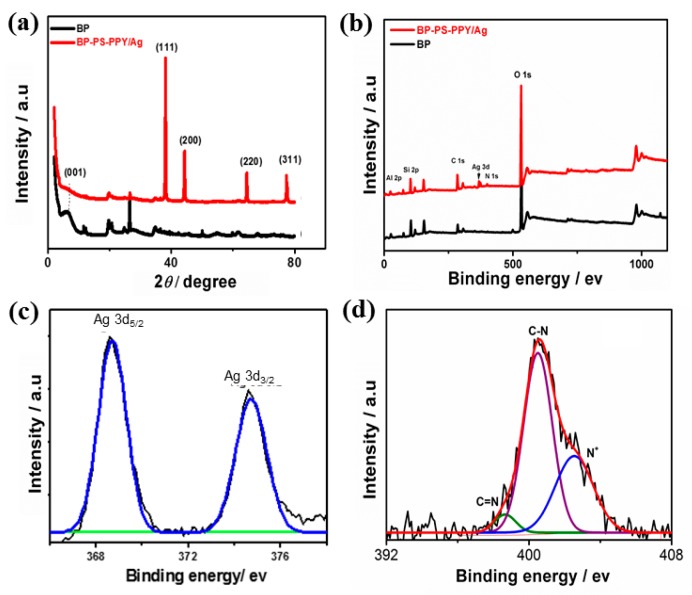
(**a**) Wide-angle X-ray diffraction (XRD) patterns and (**b**) X-ray photoelectron spectroscopy (XPS) survey of BP-Ps-PPy/Ag and BP. (**c**) High-resolution XPS spectra of (**c**) Ag and (**d**) N.

Figure 3a shows the TEM image of typically formed Ag-free BP-PS, which was formed in multi-layers of sheet-like nanostructures (Figure 3a). The high-magnification TEM show that, the as-made nanosheets were not exfoliated and with a slight agglomeration (Figure 3b). Intriguingly enough, the nanosheet morphology of BP-PS was fully preserved after the photopolymerization process in the presence of PPy and AgNO_3_ (Figure 3c). Meanwhile, the as-obtained BP-PS-PPy nanosheets were highly exfoliated without any noticed aggregation. This is attributed to anchoring of Ag NPs on N-atoms of PPy, thus precluding the agglomeration of the as-formed nanosheets during the polymerization step. Mono-dispersed Ag NPs with an average diameter of 82 nm were well distributed on the surface of the BP-PS-PPy nanosheets (Figure 3d). This is mainly attributed to the great reduction power of PPy monomer towards AgNO_3_ under UV-light irradiation. The crystalline structure of the as-made BP and BP-PS-PPy/Ag nanoarchitectures is investigated by the XRD analysis (Figure 4a). The average crystallite size of AgNPs estimated through XRD (using Scherrer’s equation) [41] is 80.9 ± 0.7 nm, which matches the size estimated by TEM.

The results show that BP displays the typical diffraction patterns with an amorphous crystalline structure as reported elsewhere [42,43]. Meanwhile, BP-PS-PPy/Ag reveals the typical, {111}, {200}, {220}, and {311} facets of face-centered cubic (*fcc*) structure of Ag (Figure 4a) [44,45]. These patterns account for the metallic nature of Ag particles produced after UV-induced reaction of Py and AgNO_3_ as judged from JCPDS file No. 00-001-1164. Interestingly, XRD patterns of Ag are slightly positively shifted relative to pure Ag NPs patterns previously reported in the literature [46], demonstrating its electronic interaction with BP-PS-PPy [47,48]. Intriguingly, BP-PS-PPyAg NPs does not display the {001} facet of BP, indicating the formation of complete exfoliated hybrid BP-PS-PPy/Ag nanocomposite in line with the TEM micrograph. XPS is used to confirm the electronic structure and surface composition of BP and BP-PS-PPy/Ag, which both show the core level of Al 2p, Si 2p, C 1s, Ag 3d, N 1s, and O 1s (Figure 3b). Table 1 reveals the surface composition evaluated by the XPS, which depicts the main elements of BP and BP-PS-PPy/Ag nanoarchitecture. The determined atomic ratios of N/Ag are about 4.69/1.6, respectively, inferring the strong affinity of PPy towards AgNO_3_. Meanwhile, the high resolved amount of C (35%) in BP-Ps-PPy/Ag is mainly originated from the multiple repeated units of PPy (C_4_H_2_N-), indicating the formation of PPy/Ag grafted BP-PS. The high-resolution spectrum of Ag 3d reveals only two main peaks of Ag 3d_5/2_ at 368.1 eV and Ag 3d_3/2_ at 374.1 eV. The absence of any oxide phases of Ag indicates the purity of the as-formed Ag NPs. The binding energies of Ag 3d are slightly blue-shifted relative to pure Ag.

This is plausibly attributed to the interaction between Ag NPs and N-atoms of PPy. The N 1s spectrum is deconvoluted to three peaks assigned to C=N, C-N, and N^+^ at 398.4, 400.3 and 401.7 eV respectively, which are the predominant peaks for PPy.

**Figure 5:**
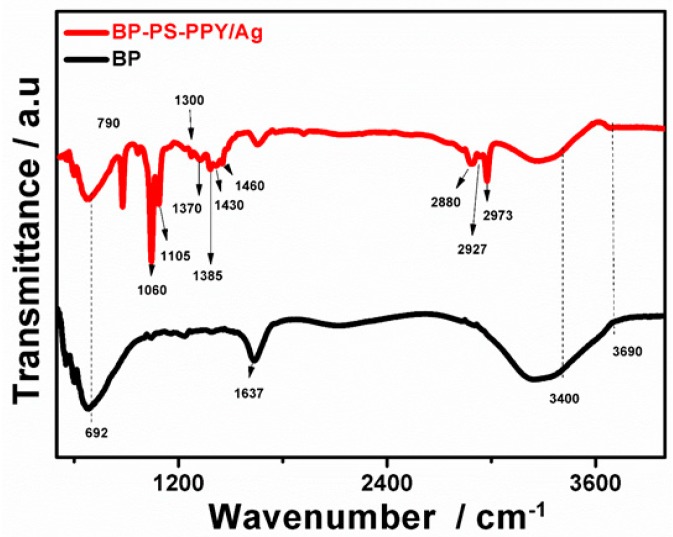
Fourier transformed infrared spectra (FTIR) analysis of typically prepared BP-Ps-PPy/Ag nanocomposite and BP.

Figure 5 displays the FTIR analysis of BP-PS-PPy/Ag and BP. The results exhibit the main characteristic peaks of BP, including the Si-O-Si (1000–1200 cm^−1^), kaolinite portions Al-OH at (3696 cm^−1^) and Si-O-Al at (692 cm^−1^) [39]. Meanwhile, BP-Ps-PPy/Ag reveals additional peaks rather than that of BP attributed to -CH_3_ (2973 cm^−1^), -CH_2_ (2926 cm^−1^), and -CH (2879 cm^−1^), which are the main stretching vibration modes of PPy [34]. Moreover, the vibration modes of C-N (1370–1460 cm^−1^), C-H (686 and 1206 cm^−1^), =C-H (1299 cm^−1^), C-H wagging vibration (786 cm^−1^), and NO_3_^−^ (1385 cm^−1^). The additional new peaks of Si-O-C (1065–1105 cm^−1^) demonstrating the chemical binding between the PS coupling agent and BP [49], confirms the successful photopolymerization of PPy with BP-PS.

Various approaches were successfully developed for precise design of PPY/Ag-based nanocomposites [50]. However, the facile synthesis of ternary BP-PS-PPy/Ag nanoarchitecture remains a significant challenge and is rarely reported to the best of our knowledge [34,35]. Meanwhile, previous reports emphasized only the multiple step reactions, which isolate between the preparation of Ag NPs and PPy. Different from these methods, our presented approach is easy, and allows the synthesis of ternary BP-PS-PPy/Ag. This is derived by the in-situ photopolymerization of PPy in the presence of BP-PS and AgNO_3_ as a photosensitizer, currently Py monomer facilitates the reduction of AgNO_3_ to form Ag NPs (Figure 1). Consequently, the as-formed PY-Ag bonded subsequently with BP-Ps, which were self-assembled to form nanosheets after the complete photopolymerization. It should be noticed that, Ag NPs prevent the agglomeration of the nanosheets during the polymerization, results in high-exfoliated nanosheets decorated with Ag NPs. This structural and compositional feature is important in electrocatalytic applications. It should be noticed that, the electroreduction activity of H_2_O_2_ on BP-Ps-PPy/Ag hybrid composite was not yet reported [35].

**Figure 6:**
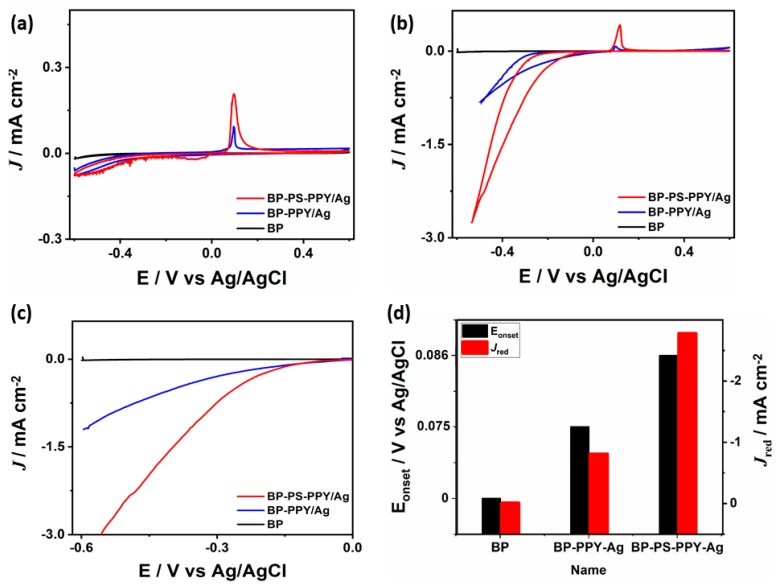
(**a**) Cyclic voltammograms (CVs) measured in an in aqueous solution of PBS (pH 7.4), at a scan rate of 50 mV s^−1^ without and (**b**) with 20 μM H_2_O_2_. (**c**) Linear sweep voltammetry measured in a solution of PBS (pH 7.4) containing 20 μM H_2_O_2_ at a scan rate of 50 mV s^−1^. (**d**) Comparison of the different *E_Onset_* and *J_Red_*.

The electrocatalytic activity of the typical prepared BP-PS-PPy/Ag nanocomposites is benchmarked relative to BP-PPy/Ag and BP towards the reduction of H_2_O_2_ detection. Figure 6a represents the CVs of the as-made catalysts measured in an aqueous solution of PBS (pH 7.4) at a scan rate of 50 mV s^−1^ with potential range (−0.6 to 0.6 V vs. Ag/AgCl). All electrocatalysts reveal the typical CVs featured including hydrogen adsorption/desorption, Ag-redox, and oxygen evolution. The capacitance currents of BP-PS-PPy/Ag and BP-PPy/Ag were significantly higher than that of metal-free BP, indicating its higher conductivity and surface area. In addition, the capacitive current increases with the surface area.

**Figure 7:**
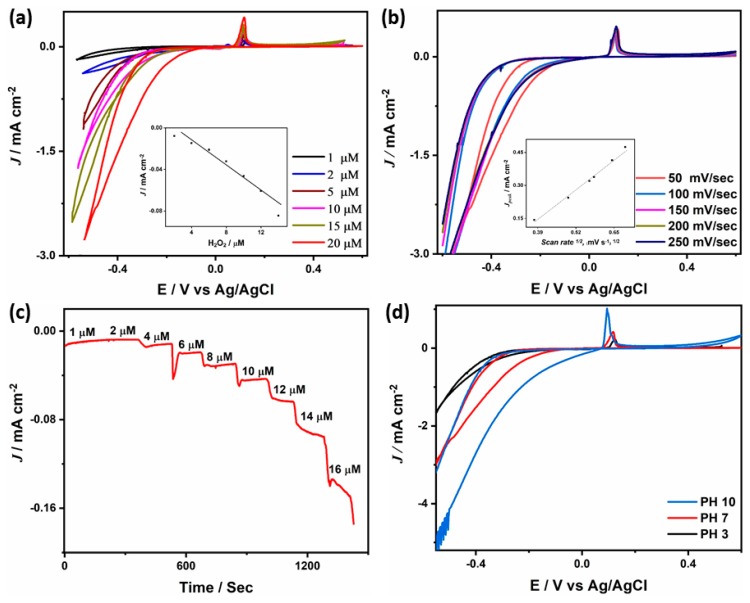
(**a**) CVs measured in an aqueous solution of (pH 7.4) containing different concetartion of H_2_O_2_ at 50 mV s^−1^ (**b**) CVs measured at diffret scan rates. The insets in (**a**) and in (**b**) show the plot of current versus H_2_O_2_ concetration and current versus scan rates, respectively. (**c**) Chronoamperometric curve, (**d**) CVs measured in solutions with different pH values in the presence of 20 μM H_2_O_2_ at 50 mV s^−1^.

Figure 6b shows the CVs measured in PBS solution containing 20 μM of H_2_O_2_ at 50 mV s^−1^ which depicts the higher H_2_O_2_ reduction activity of BP-PS-PPy/Ag compared with its counterpart BP-PPy/Ag and BP nanostructures. BP-PS-PPy/Ag produces a higher current density under any applied potential than that from the BP-PPy/Ag, as can be seen from the linear sweep voltammograms shown in Figure 6c. This is owing to the combination of BP-PS-PPy and Ag NPs, which enhances the electrical conductivity and provide more active sites for H_2_O_2_ reduction. In this regard, the H_2_O_2_ reduction current (*J*_red_) on BP-PS-PPy/Ag (−2.78 mA cm^−2^) is around three times higher than that on BP-PPy/Ag (−0.81 mA cm^−2^) (Figure 6d), inferring the potent electrocatalytic behavior of BP-PS-PPy/Ag. This is evidenced in the positive onset reduction potential (*E_onset_*) on BP-PS-PPy/Ag (0.075 V) compared with that on BP-PPy/Ag (0.086 V), owing to its higher conductivity (Figure 6d). This indicates the fast reduction kinetics on BP-PS-PPy/Ag. The CVs were measured on BP-Ps-PPy/Ag in an aqueous solution of PBS (pH 7) containing 20 μM H_2_O_2_ at different scan rates (Figure 7b). It is obvious that the reduction current peak increased steadily upon increasing the scan rate from 50 to 250 mV s^−1^, inferring the the quick mass trsnafre on BP-PS-PPy/Ag. The corresponding plot of the peak current versus the square root of the scan rate displayed a linear relationship.

Figure 7a shows that the H_2_O_2_ reduction currents is proportionally enhanced with increasing the concentration of H_2_O_2_ from 1 μM to 20 μM. Moreover, chronoamperometry study (*J*-*T* in Figure 7c) which is done by applying 0.2 V on BP-PS-PPy/Ag electrode with adding different concentrations of H_2_O_2_ during maintaining PBS in a stirring condition shows a rapid and sensitive response to H_2_O_2_ as the response current increases with increasing the H_2_O_2_ concentration and the current plataeu is achieved in 2 s. As developing an efficient catalyst for electrocatalytic reduction of H_2_O_2_ over wide ranges of pH is a significant challenge, the H_2_O_2_ reduction activity on BP-Ps-PPy/Ag nanoarchitectures was investigated under different pH values ranged from 3 to 10 (Figure 7d). BP-Ps-PPy/Ag is found to be able to reduce H_2_O_2_ under diffter pH values (3, 7, and 10). The reduction current achieved at a pH of 10 (–5.03 mA cm^−2^) is almost 2-and 4-fold higher than that at a pH of 7 and 3, respectively. This is owing to the fast kinetics of H_2_O_2_ reduction under alkaline conditions. It should be noticed that under acidic conditions we could not resolve any additional peak for the oxidation of Ag, indicating its stability against corrosion. This can be attributed to the strong electronic interaction between BP-PS-PPy and Ag NPs, which stabilizes Ag against dissolution under acidic conditions. The durability of the electrocatalysts is a decisive factor in large-scale applications. The stability of the typically prepared BP-PS-PPy/Ag nanoarchitectures is investigated via benchmarking the chronoamperometric current-time with a consecutive addition of H_2_O_2_ into PBS (pH 7.4) at an applied potential of (−0.3 V). The results reveal the rapid chronoamperometric responses of BP-PS-PPy/Ag to changing the concentrations of H_2_O_2_ along with typical steady-state current rising until reach the stable value. Interestingly, BP-Ps-PPy/Ag achieved a quick current response at 1 μM within 1 s, which is superior to previously reported for Fe_3_O_4_/PPy/Ag nanocomposite (5 s), PPy nanofiber-AgNPs-rGO (3 s), and PPyNPT-Ag (3 s) [51,52,53]. The calibration curves depict that the linear detection ranges of H_2_O_2_ ranged from 1 μM to 20 μM with a detection limit of 1 μM. The as-synthesized BP-PS-PPy/Ag showed a detection limit of H_2_O_2_ detection lower than that of beforehand reported 1 μM and 1.7 μM and 1.8 μM [51,52,53].

**Figure 8:**
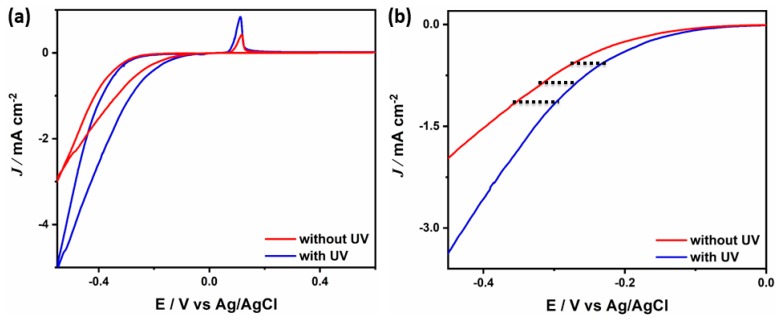
(**a**) CVs and (**b**) Linear sweep voltammetry measured in a solution of PBS (pH 7.4) containing 20 μM H_2_O_2_ at a scan rate of 50 mV s^−1^ in the presence and in the absence of UV-light irradiation.

The H_2_O_2_ reduction performance on BP-Ps-PPy/Ag, is measured under the UV-visible light irradiation to further sort out the catalytic effect of Ag NPs (Figure 8a). Interestingly, the H_2_O_2_ reduction current on BP-Ps-PPy/Ag under light (5.3 mA cm^−2^) is almost double its counterpart measured in the dark (2.8 mA cm^−2^). Meanwhile, the *E*_onset_ under light is significantly more positive than that under dark at any applied potential point as indicated by the dashed line in Figure 8b. This implies the more facile reduction kinetics under light, ascribed to the inbuilt optical properties of Ag NPs, which produce photo-generated electrons and release them to the BP-Ps-PPy.

These findings clearly display the superior electrocatalytic H_2_O_2_ reduction activity of BP-PS-PPy/Ag nanoarchitecture than that of BP-PPy/Ag and BP. This is attributed to the combination between the remarkable physicochemical properties of BP-PS-PPy and outstanding catalytic and optical properties of Ag NPs [54,55]. Mainly, the exfoliated BP-PS-PPy nanosheets provide various adsorptions and active sites for H_2_O_2_, which maximize the utilization of Ag NPs during H_2_O_2_ reduction [56,57]. Meanwhile, the synergistic and electronic interaction between Ag NPs and BP-PS-PPy originates various accessible active sites for H_2_O_2_ reduction as well as produces oxygenated species which accelerates the reduction kinetics at low overpotentials.

Table 2 compares the performances of the actual electrocatalyst to those of similar ones prepared in different conditions. The as-prepared BP-PS-PPY/Ag present the lowest detection limit.

## 4. Conclusions

To sum up, a facile, versatile, low-cost and scalable roadmap is presented for controlled synthesis of BP-PS-PPy/Ag nanoarchitectures. This is simply based on the in-situ photopolymerization of pyrrole in the presence of BP-PS and AgNO_3_ as a photosensitizer. Meanwhile, pyrrole facilitated the in-situ reduction of silver nitrate to form Ag NPs. The as-produced nanoarchitecture is assembled in well-defined exfoliated BP-PS-PPy nanosheets decorated with monodispersed Ag NPs. The detection limit of H_2_O_2_ in the presence of BP-Ps-PPy/Ag was about 1 µM as well as a fast response of 1 s. Currently, BP-PS-PPy/Ag is found to be an efficient catalyst for H_2_O_2_ reduction over wide ranges of pH, ranging from 3 to 10. Moreover, the UV-light irradiation enhanced the photocatalytic H_2_O_2_ reduction activity of BP-PS-PPy/Ag, owing to the optical properties of Ag NPs. The presented method may open new windows towards usage of prepared BP-PS-PPy/Ag for various catalytic reactions.

## Figures and Tables

**Table 1:** Elemental composition determined by XPS.

Catalysts	Si	Al	O	C	N	Ag	Na	K	Ca
BP	21.4	7.9	60.3	7.00	-	-	2.70	0.21	0.50
BP-PS-PPy/Ag	15.2	4.6	38.3	35.0	4.69	1.60	traces	0.30	0.40

**Table 2:** Summary of the polypyrrole-based composites on various kinds of supports for H_2_O_2_ detection.

Support/Substrate	Material	Experimental Details	Limit of Detection	References
Bentonite Clay	PPy-Ag coating	UV- induced polymerization using AgNO_3_ as oxidant, light intensity ~28 mW/cm^2^	1 µM	This work
Graphene oxide	PPy-Ag nanofibers-silver nanoparticles	Electropolymerization on the surface of the modified electrode through amperometry process	1 mM	[58]
Glassy carbon electrodes	PPy-Silver Nanostrip Bundles	Chemical oxidative polymerization using AgNO_3_ as oxidant, Time ~90 min	43.60 μM	[59]
Glassy carbon electrode	Ag nanoparticle-decorated polypyrrole colloids	Chemical oxidative polymerization using AgNO_3_ as oxidant, Time ~90 min	90 mM	[60]
Fe_3_O_4_ spheres	PPy coating	Chemical oxidation polymer-ization using FeCl_3_ as the oxidant under the assistance of an anion surfactant.	1.6 µM	[61]
Natural biomineralization hydroxyapatite (BioHAP)	PPy-(AgHg) coating	Chemical oxidation polymer-ization using ammonium persulfate (APS) as an oxidant in the presence of BioHAP as a substrate PPy/APS = 1:0.2; Time ~48 h	0.27 mM	[62]
(NiO) nikel oxide	PPy-NiO Needle like nanocomposites	Chemical oxidation polymer-ization in the presence of NiO composite in the presence of hydrazine PPy/hydrazine = 0.5/0.01 The temperature was 60 °C, 60 min	5.77 mM	[63]
